# Detection and Quantification of Tp53 and p53-Anti-p53 Autoantibody Immune Complex: Promising Biomarkers in Early Stage Lung Cancer Diagnosis

**DOI:** 10.3390/bios12020127

**Published:** 2022-02-16

**Authors:** Keum-Soo Song, Satish Balasaheb Nimse, Shrikant Dashrath Warkad, Jung-Hoon Kim, Hey-Jin Kim, Taisun Kim

**Affiliations:** 1 Biometrix Technology, Inc., 2-2 Bio Venture Plaza 56, Chuncheon 24232, Korea; hanlimsk@empas.com (K.-S.S.); shrikant.warkad@gmail.com (S.D.W.); jhkim@bmtchip.com (J.-H.K.); 2 Institute of Applied Chemistry and Department of Chemistry, Hallym University, Chuncheon 200702, Korea; satish_nimse@hallym.ac.kr; 3 Department of Laboratory Medicine, Korea Cancer Center Hospital, Korea Institute of Radiological and Medical Sciences, Seoul 01812, Korea; heyjin@kirams.re.kr

**Keywords:** stage I, lung cancer, NSCLS, SCLC, p53, autoantibody, biomarkers, DAGON method

## Abstract

Lung cancer is a leading cause of death worldwide, claiming nearly 1.80 million lives in 2020. Screening with low-dose computed tomography (LDCT) reduces lung cancer mortality by about 20% compared to standard chest X-rays among current or heavy smokers. However, several reports indicate that LDCT has a high false-positive rate. In this regard, methods based on biomarker detection offer excellent potential for developing noninvasive cancer diagnostic tests to complement LDCT for detecting stage 0∼IV lung cancers. Herein, we have developed a method for detecting and quantifying a p53-anti-p53 autoantibody complex and the total p53 antigen (wild and mutant). The LOD for detecting Tp53 and PIC were 7.41 pg/mL and 5.74 pg/mL, respectively. The detection ranges for both biomarkers were 0–7500 pg/mL. The known interfering agents in immunoassays such as biotin, bilirubin, intra-lipid, and hemoglobin did not detect Tp53 and PIC, even at levels that were several folds higher levels than their normal levels. Furthermore, the present study provides a unique report on this preliminary investigation using the PIC/Tp53 ratio to detect stage I–IV lung cancers. The presented method detects lung cancers with 81.6% sensitivity and 93.3% specificity. These results indicate that the presented method has high applicability for the identification of lung cancer patients from the healthy population.

## 1. Introduction

Lung cancer is a leading cause of death worldwide, claiming nearly 1.80 million lives in 2020 [1]. The overall 5-year relative survival rate for lung cancer is 21% [2,3]. However, if diagnosed at a localized stage, the 5-year survival rate is 59%. Unfortunately, only 17% of lung cancers are diagnosed at a localized stage [4]. Screening with low-dose computed tomography (LDCT) reduced lung cancer mortality by about 20% compared to standard chest X-rays among current or heavy smokers [5]. The American Cancer Society recommends annual LDCT-based lung cancer screening in apparently healthy patients (aged 55 to 80 years) who have at least a 30 pack-year smoking history, current smokers, and those who have quit smoking within the past 15 years [6]. However, several reports indicate that LDCT has a high false-positive rate [7]. According to a report, about 25% of subjects are classified as being positive for lung cancer after three rounds of LDCT screening. Unfortunately, 96% of these subjects were confirmed as being false positives upon biopsy [8]. Therefore, a complementary test that can be combined with LDCT to improve the diagnostic capacities and to reduce false-positive rates is highly awaited [9,10,11]. In this regard, methods based on biomarker detection offer excellent potential for developing noninvasive cancer diagnostic tests to complement LDCT for detecting stage 0∼IV lung cancers [12].

The wild-type p53, a short-lived (half-life of ~5–20 min) tumor suppressor protein, is found in most cell types [13]. The missense mutations that produce a stable form of the mutant p53 protein (half-life of 12–>30 h) are the most common alterations in human cancers, including in lung cancer [14,15,16]. The overexpression and accumulation of stabilized mutant p53 proteins induce a specific humoral response in cancer patients, leading to the release of the anti-p53 autoantibody (p53-AAb) [17]. The p53-AAbs and mutant p53 protein are found in the plasma of over 60∼70% of lung cancer patients [18]. Notably, p53-AAb is extremely rare in the plasma of the healthy population [19]. Therefore, developing a noninvasive serological test for detecting p53 autoantibodies can allow for the highly specific detection of early-stage lung cancer [20,21,22]. It is also crucial to note that the healthy population only exhibits wild-type p53. However, both wild- and mutant-type p53 proteins co-exist in the plasma of lung cancer patients. Therefore, we hypothesized that p53-Anti-p53-Autoantibody immune complex (PIC) levels would be higher in lung cancer patients compared to in healthy populations. Additionally, we hypothesized that the total p53 (Tp53: wild-type and mutant p53) would be lower in lung cancer patients than it would be in healthy people. Hence, the PIC/Tp53 ratio would be several folds larger in cancer patients than it would be in healthy people, allowing for early stage lung cancer to be diagnosed with high specificity.

Herein, we present a method for detecting and quantifying Tp53 and PIC in plasma samples. The LOD for detecting Tp53 and PIC were 7.41 pg/mL and 5.74 pg/mL, respectively. The detection ranges for both biomarkers were 0–7500 pg/mL. The known interfering agents in the immunoassays such as biotin, bilirubin, intra-lipid, and hemoglobin did not detect Tp53 and PIC, even at levels that were several folds higher levels than their normal levels. Furthermore, the present study provides a unique report on the preliminary investigation on the use of the PIC/Tp53 ratio to detect stage I–IV lung cancers. The method that is presented here detects lung cancers with 81.6% sensitivity and 93.3% specificity. These results indicate that the presented method has high applicability for the identification of lung cancer patients from the healthy population.

## 2. Materials and Methods

### 2.1. Materials

Required chemicals, including hemoglobin and biotin, were procured from Sigma-Aldrich Chemicals (Seoul, South Korea). All amine-containing oligonucleotides (NH_2_-DNA) were obtained from Bioneer (Daejeon, South Korea). Mouse anti-human p53 capture antibody (Catalog #. SC-126) and mouse anti-human p53 detection antibody (Catalog #. SC-393031) were purchased from Santa Cruz Biotechnology, Inc. (Dallas, TX, USA). Goat anti-human IgG (Catalog# ABIGG-0500) and goat anti-mouse IgG (Catalog# ABGAM-0500) were purchased from Arista Biologicals Inc. (Allentown, PA, USA). The p53 antigen (Catalog #. ab237007) was purchased from Abcam (USA). The oligonucleotides that were used in this study were procured from Bioneer (Daejeon, South Korea). Fluorescent beads that were 0.2 um in size (FB, λ_ex_ = 622 nm, λ_em_ = 645 nm, Catalog #. F8806) and that had been modified with carboxyl functional groups were purchased from Thermo Fisher Scientific (Waltham, MA, USA).

### 2.2. Clinical Samples

From 17 July 2020 through 31 December 2020, about 15 healthy individuals and 30 lung cancer patients were enrolled in the study. Biopsy-positive stage I to IV lung cancer patients were chosen for this study. Of the 30 lung cancer patients, 23 were identified as having non-small cell lung cancer (NSCLC; stage I = 7, stage II = 6, stage III = 5, stage IV = 5), and 7 were identified as having small cell lung cancer (SCLC; limited disease = 4, extended disease = 3). Plasma samples were collected before the beginning of treatment or before the surgical removal of the tumor. The Ethical Clearance Committee on Human Rights Related to Research Involving Human Subjects of Korea Cancer Central Hospital, Korea Institute of Radiological & Medical Sciences, Nowon-Gu, Seoul, South Korea, approved this study (KIRAMS 2020-06-017-002).

### 2.3. Preparation of Bio-Conjugates

#### 2.3.1. Synthesis of p53-cAb-DNA Conjugate

According to our previous reports, the p53-cAb-DNA conjugates were prepared in three steps [23,24]. First, p53-cAb was reacted with 2-iminothiolane in bicarbonate buffer to produce thiol-functionalized p53-cAb [25]. In a second step, reacting amine-functionalized DNAs with the sulfo-SMCC resulted in the formation of the DNA-sulfo-SMCC conjugate. Finally, reacting the thiol-functionalized p53-cAb with the DNA-sulfo-SMCC in 1X PBS buffer solution resulted in the formation of the p53-cAb-DNA conjugate.

#### 2.3.2. Synthesis of Anti-Mouse IgG-Cy5 and Anti-Human IgG-Cy5

The manufacturer’s (GE Healthcare UK Limited, Buckinghamshire, UK) standard reaction protocol for tagging biomolecules with fluorescent cyanine-5 dye (Cy5) was used to synthesize the anti-mouse IgG-Cy5 conjugates. In brief, the amine functions in the IgG were allowed to react with the monofunctional NHS-ester Cy5Dye^TM^ to obtain Cy5-labeled goat anti-mouse IgG (anti-mouse IgG-Cy5). Cy5-labeled goat anti-human IgG were synthesized by following the same method as the one described for the synthesis of anti-mouse IgG-Cy5.

#### 2.3.3. Synthesis of p53-dAb-FB and Anti-Human-IgG-FB Conjugates

The p53-dAb and anti-human-IgG were labeled with FB by following the reported method [26]. First, we used the 1-ethyl-3-(3-dimethylaminopropyl) carbodiimide hydrochloride (EDC) to activate the carboxyl groups on the FB (activated-FB). The activated FBs were then reacted with p53-dAb to produce p53-dAb-FB conjugates. The anti-human-IgG-FB conjugates were also prepared following a similar method.

#### 2.3.4. Synthesis of FB-DNA Conjugate

The NH_2_-DNA were reacted with the activated-FB in 1X PBS buffer to obtain the FB-DNA conjugates. The DNA sequence in the FB-DNA conjugate was complementary to the DNA immobilized on the hybridization control (HC) line.

### 2.4. Preparation of Lateral Flow Strip Membranes (LFSM) for the Detection of PIC and Tp53

The LFSMs used to detect PIC and Tp53 were prepared according to the previously reported method [27,28,29,30]. In brief, 18 pmol/μL solutions containing probe DNAs corresponding to the test line and hybridization control (HC) line were lined on the 9G membranes and allowed to immobilize. The membranes were then soaked in the blocking solution after the immobilization step and were dried to generate 9G DNA membranes. The obtained 9G membranes were used to manufacture LFSM.

### 2.5. Quantification of PIC in the Lung Cancer Sample Using 9G DNAChip and DAGON Method

The quantification of PIC in a lung cancer sample using a 9G DNAChip and the DAGON method is briefly explained in the following paragraphs.

#### 2.5.1. Obtaining a Standard Curve for PIC Quantification

As shown in Figure S1a, a standard curve was obtained by mixing 0.5 µg/mL of p53 with various concentrations of the mouse origin detection antibody (dAb, 5000.0, 2500.0, 1250.0, 625.0, 312.0, 0 pg/mL) to obtain the p53-dAb complexes, which resembled the PIC found in lung cancer samples. The obtained complexes were mixed with the hybridization solution containing p53-cAB-DNA conjugate and anti-mouse-IgG-Cy5 (1 µg/mL). Then, loading 60 µL of these solutions on the 9G DNAChips was followed by incubation for 30 min at 25 °C. The chips were washed and dried using a commercial centrifuge (1000 rpm). Finally, the chips were scanned and analyzed using ScanArrayLite and Quant Array software. Experiments were performed in triplicate, and the standard curve was obtained using the average values as shown in Figure S1c.

#### 2.5.2. Quantification of PIC in Lung Cancer Sample and its Quantification

As shown in Figure S1b, lung cancer plasma sample (20.0, 10.0, 5.0, 2.5, 1.2, and 0 µL) was added to a hybridization solution containing p53-cAb-DNA conjugate and anti-human IgG-Cy5 (1 µg/mL). The 60 µL of these solutions were loaded in each chamber of the 9G DNAChip and then incubated at 25 °C for 30 min. The 9G DNAChip was then rinsed with washing buffer solutions A and B successively for 2 min each and then dried with a commercial centrifuge (1000 rpm). The fluorescence signal on the 9G DNAChips was measured with ScanArrayLite, and the images were analyzed using Quant Array software. As shown in Figure S1b, the fluorescence intensity of 38,138 was observed for the 10 µL lung cancer plasma sample. By extrapolating this fluorescence intensity in the standard curve presented in Figure S1c, the lung cancer sample was found to contain 1250.02 pg/mL of PIC. Hence, this lung cancer sample containing 1250.0 pg/mL of PIC was used as the standard for further experiments.

### 2.6. General Procedure for the Detection of PIC and Tp53 Using LFSM

#### 2.6.1. Detection of Tp53 Using LFSM

For Tp53 detection, a 20 µL serum sample was incubated with the 100 µL of the solution containing the p53-cAb-DNA conjugate (10 fmol/mL), p53-dAb-FB conjugate (0.07 fmol/mL), and FB-DNA (10 fmol/mL) in an e-tube and incubated at 25 °C for 10 min in a thermo-controller. After the incubation step, 60 µL of reaction buffer containing FB-DNA complementary to the DNA immobilized on the hybridization control (HC) line as added to the reaction tube. Then, the reaction mixture was loaded on the LFSM and allowed to hybridize for 10 min at 25 °C. The highly specific DNA–DNA hybridization allowed for the capture of the p53-dAb-FB-Tp53-p53-cAb-DNA biomolecular complexes and the Cy5-DNA on the test line and HC line, respectively. The unbound materials were then washed at 25 °C in a washing step that was 10 min long by loading 170 mL washing solution (0.1% SDS in 4× SSC, pH = 7.4). After the washing step, the LFSMs were scanned in the BMT Reader^TM^ to obtain the results.

#### 2.6.2. Detection of PIC Using LFSM

For PIC detection, a 20 µL serum sample was incubated with with 100 µL of the solution containing the p53-cAb-DNA conjugate (10 fmol/mL), anti-human-IgG-FB conjugate (0.07 fmol/mL), and FB-DNA (10 fmol/mL) in an e-tube and incubated at 25 °C for 10 min in a thermo-controller. After the incubation step, 60 µL of reaction buffer was added to the reaction tube, and then the whole reaction mixture was loaded on one side of the LFSM and allowed to hybridize for 10 min at 25 °C. The highly specific DNA–DNA hybridization allowed for the capture of the anti-human-IgG-FB-PIC-p53-cAb-DNA biomolecular complexes and the Cy5-DNA on the test line and HC line, respectively. The unbound materials were then washed at 25 °C in a washing step that was 10 min long by loading 170 mL of washing solution (0.1% SDS in 4× SSC, pH = 7.4). After the washing step, the LFSMs were scanned in the BMT Reader^TM^ to obtain the results.

### 2.7. Optimization of Incubation Time, Hybridization Time, and Washing Time for Tp53 and PIC Detection

The standard samples containing Tp53 (1250 pg/mL) and PIC (1250 pg/mL) were used in the serial dilution test to optimize the incubation time, hybridization time, and washing time. The levels of PIC and Tp53 in each serially diluted sample were determined, and the obtained data were used for linear regression analysis to determine the linearity coefficient (R^2^). The R^2^ value > 0.99 was used to identify the optimized incubation time, hybridization time, and washing time. To optimize the incubation time, the general procedures for detecting Tp53 and PIC were used, except for the incubation step. The incubation was carried out at 25 °C in a thermo-controller for 5, 10, 20, and 30 min by keeping the 10 min hybridization and washing steps constant for each measurement. The hybridization step was performed at 25 °C for 5, 10, 20, and 30 min to optimize the hybridization time. The incubation and 10 min washing steps were kept constant for each measurement. The washing time was optimized by performing the washing step at 25 °C for time intervals, including 5, 10, 20, and 30 min intervals, while keeping the incubation and hybridization steps at 10 min each. Each measurement was performed in triplicate.

### 2.8. Interference Study 

The possible interference effect of blood components on PIC detection and Tp53 was evaluated by spiking the plasma samples with biotin, bilirubin, intra-lipid, and hemoglobin. The plasma sample containing PIC (1250 pg/mL) and the plasma sample containing Tp53 (1250 pg/mL) were spiked with biotin (3.0 µg/mL), bilirubin (0.2 mg/mL), intra-lipid (0.20%), and hemoglobin (1.0 mg/mL). The PIC and Tp53 levels were measured in the serially 1/2-diluted spiked samples. All measurements were performed in triplicate.

### 2.9. Linearity Study Using Clinical Samples, Standard Curves for Tp53 and PIC

The linearity in the serial dilution test was evaluated by serially diluting clinical plasma samples from healthy controls (n = 3) and lung cancer patients (n = 3). The Tp53 and PIC levels were measured in the diluted samples and used for the linear regression analysis. Each experiment was performed in triplicate. Standard curves for Tp53 and PIC were obtained by diluting a stock solution in analyte-free human plasma (8 calibration points; 0–7500 pg/mL). The stock solution for Tp53 was obtained by dissolving the contents in the sample vial as per the manufacturer’s protocols. The PIC stock solution was obtained by diluting the lung cancer plasma samples. The mean values (n = 3) for each calibration point were used to construct the respective standard curves.

### 2.10. Statistical Analysis

Data are expressed as the mean with interquartile range (IQR), whereas the categorical data are given in counts and percentages. We used Medcalc 17.4.4 (Medcalc Software, Mariakerke, Belgium) for the statistical analysis of the clinical analysis and OriginPro^®^ 2020 software (OriginLab Corporation, Northampton, USA) for the graphing and linear regression analyses. The clinical study results are presented as sensitivity and specificity at a 95% confidence interval.

## 3. Results and Discussion

**Scheme 1:**
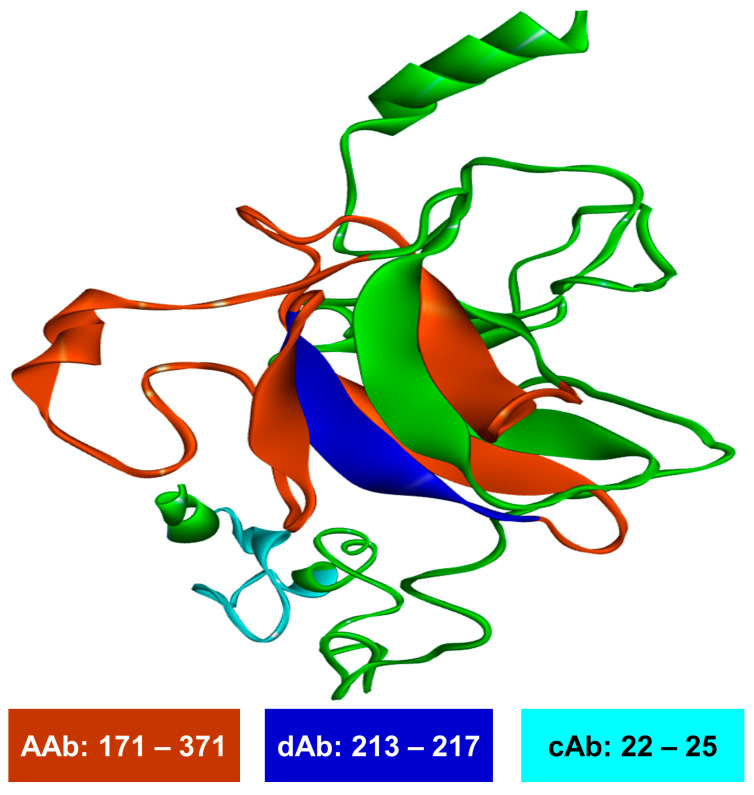
Total sequence of the p53 antigen marked for the autoantibody (AAb:171–371), detection antibody (dAb:213–217), and capture antibody (cAb:22–25) binding domains.

There are several reports on the quantification of mutant p53 proteins in samples from lung cancer patients. However, the detection of wild-type p53 is seldom used to diagnose lung cancer. Even though the wild-type p53 protein has a short half-life, it is present at high levels in the clinical samples because almost all cells express it. Therefore, the detection of the Tp53 antigen, which includes both wild and mutant types, was one of the goals of the proposed method. Hence, to do that, we studied the three-dimensional structure of the p53 protein as presented in Scheme 1. The human p53 protein contains 393 amino acids, which are divided into four major domains, including a transcriptional activation domain (aa1–aa 42), sequence-specific DNA-binding domain (aa102–aa292), oligomerization domain (aa323–aa356), and a regulatory domain (aa 360–aa393) [31].

As shown in Scheme 1, the autoantibody (AAb) dining site (aa171–aa371) overlaps with the detection antibody (dAb) binding site (aa213–aa217). The binding site (aa22–aa25) for the capture antibody (cAb) was well separated from these two binding sites. About 80–90% of the tumor mutations were found in the aa102–aa292 region [32,33]. It is important to note that the region where the major mutations are found in the mutant-type p53 overlaps with the AAb binding region. Therefore, regardless of whether it is wild-type or mutant-type p53, the anti-p53-autoantibody binds to both and forms PIC. The dAb used here can bind to free wild-type p53 and mutant-type p53, allowing us to determine the Tp53 levels in the plasma samples.

**Scheme 2:**
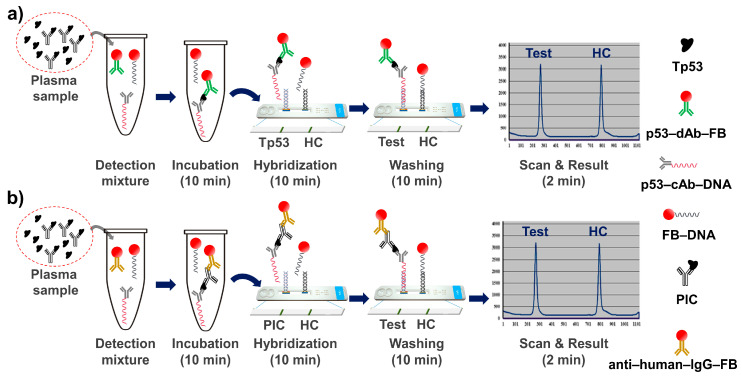
General procedure for detecting (**a**) Tp53 and (**b**) p53-anti-p53-autoantibody complex (PIC) in the plasma samples.

Scheme 2 depicts the general method for detecting PIC and Tp53 using the LFSM. The LFSMs were manufactured using the 9G DNA membranes containing the test line and an HC line. As shown in Scheme 2a, Tp53 was detected by incubating 20 μL of a plasma sample with a reaction mixture containing p53-dAb-FB, p53-cAb-DNA, and FB-DNA. As shown in Scheme 2b for PIC detection, the reaction mixture containing anti-human-IgG-FB, p53-cAb-DNA, and FB-DNA was incubated with 20 μL of a plasma sample. The incubation of these solutions allows for the formation of respective biomolecular complexes such as PIC (anti-human-IgG-FB-PIC-p53-cAb-DNA and p53-dAb-FB-Tp53-p53-cAb-DNA). After loading these solutions on the LFSM, the biomolecular complexes were captured on the test line. The FB-DNAs were captured on the HC line during the hybridization step. The loading of the washing solution on the LFSM removes the unbound biomolecular complexes, and the bound ones are then detected and quantified by scanning in the BMT Reader™.

The unavailability of commercial PIC prompted us to use the lung cancer sample as a source of PIC. We used the 9G DNAChip and DAGON method [34,35,36] to determine the PIC concentration in the lung cancer sample (see the supporting information Figure S1). The lung cancer sample contained 1250 pg/mL PIC, and it was used as a standard to develop a PIC detection method.

The serial dilution tests were performed to optimize incubation time, hybridization time, and washing time at various time intervals (5, 10, 20, and 30 min) to efficiently quantify the Tp53 and PIC in the plasma samples. As shown in Figure S2, the R^2^ values for the 5, 10, 20, and 30 min incubation times were 0.984, 0.997, 0.981, and 0.9875. Therefore, the 10 min incubation time was considered to be the optimum incubation time because it had the best linearity coefficient. Similarly, as shown in Figures S3 and S4, the R^2^ values for the 10 min hybridization time (R^2^ = 0.990) and 10 min washing time (R^2^ = 0.998) were the best among studied hybridization and washing times. As shown in Figures S5–S7 for PIC detection, the R^2^ values for the 10 min of incubation (R^2^ = 0.994), hybridization (R^2^ = 0.991), and washing time (R^2^ = 0.996) steps were the best among the other studied time intervals, for which the R^2^ values were in the range of 0.953–0.980. Hence, 10 min each of incubation, hybridization, and washing steps were considered optimum for Tp53 and PIC detection and were used in further experiments.

**Figure 1:**
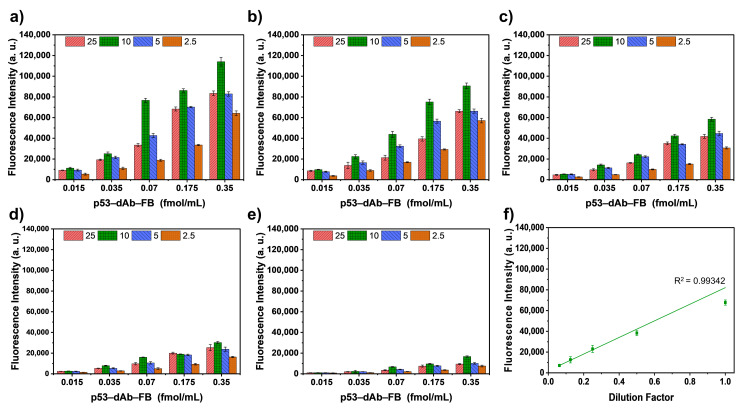
Optimization of p53-dAb-FB (0.015, 0.035, 0.07, 0.175, and 0.35 fmol/mL) and p53-cAb-DNA (2.5, 5, 10, and 25 fmol/mL) concentrations for Tp53 detection. The Tp53 (pg/mL) levels were (**a**) 5000, (**b**) 2500, (**c**) 1250, (**d**) 625, (**e**) 312; (**f**) linearity in the serial dilution of 5000 pg/mL of Tp53 using 0.07 fmol/mL of p53-dAb-FB and 10 fmol/mL of p53-cAb-DNA.

The ideal concentrations of p53-dAb-FB and p53-cAb-DNA for Tp53 detection were optimized using five Tp53 samples containing 312, 625, 1250, 2500, and 5000 pg/mL. Figure 1 depicts the data for Tp53 detection (312–5000 pg/mL) using various p53-dAb-FB (0.015, 0.035, 0.07, 0.175, and 0.35 fmol/mL) and p53-cAb-DNA (2.5, 5, 10, and 10 fmol/mL) levels.

The original plasma sample containing 5000 pg/mL of Tp53 was serially diluted with a ½ dilution factor, as shown from Figure 1a to Figure 1e. Figure 1a–e depicts that the increase in the p53-dAb-FB increases fluorescence intensity in all cases. However, an increase in the p53-cAb-DNA concentration does not show uniformity in the increase in the fluorescence signal. Specifically, as depicted in Figure 1f (Figure S8), the 10 fmol/mL of the p53-cAb-DNA and any combination of p53-dAb-FB (0.015, 0.035, 0.07, 0.175, and 0.35 fmol/mL) show a linear increase in the fluorescence intensity. We observed the linearity coefficient for the serial dilution of 5000 pg/mL of Tp53 using p53-dAb-FB (0.07 fmol/mL) and p53-cAb-DNA (10 fmol/mL) of 0.993. Therefore, 0.07 fmol/mL and 10 fmol/mL of p53-dAb-FB and p53-cAb-DNA, respectively, were considered optimum Tp53 detection in the plasma samples.

**Figure 2:**
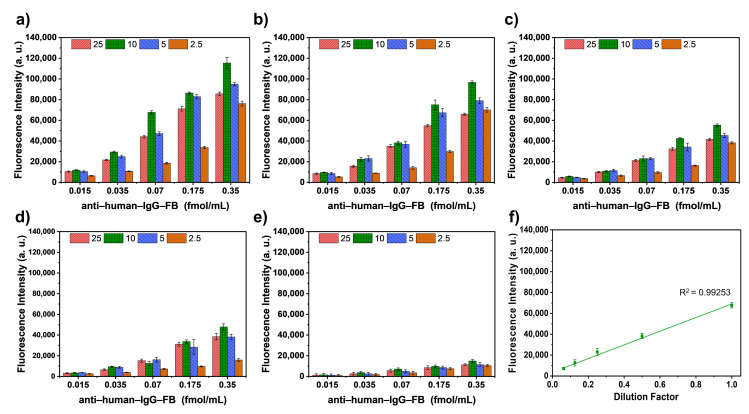
Optimization of p53-cAb-DNA (2.5, 5, 10, and 25 fmol/mL) and anti-human-IgG-FB (0.015, 0.035, 0.07, 0.175, and 0.35 fmol/mL) concentrations for PIC detection. The PIC (pg/mL) levels were (**a**) 5000, (**b**) 2500, (**c**) 1250, (**d**) 625, (**e**) 312; (**f**) linearity in the serial dilution of 5000 pg/mL of PIC using 0.07 fmol/mL of anti-human-IgG-FB and 10 fmol/mL of p53-cAb-DNA.

The concentrations of anti-human-IgG-FB and p53-cAb-DNA for PIC detection were optimized using the lung cancer sample containing 5000 pg/mL of PIC. Solutions containing various combinations of anti-human-IgG-FB (0.015, 0.035, 0.07, 0.175, and 0.35 fmol/mL) and p53-cAb-DNA (2.5, 5, 10, and 10 fmol/mL) were mixed the various serially diluted PIC samples (5000, 2500, 1250, 625, 312). The obtained results are presented in Figure 2.

As shown in Figure 2, the lung cancer plasma sample containing 5000 pg/mL of PIC was serially half diluted, and the corresponding fluorescence intensities in the presence of various concentrations of anti-human-IgG-FB and p53-cAb-DNA were measured. Figure 2a–e depicts how the anti-human-IgG-FB concentration increases the fluorescence intensity for all measurements. Interestingly, the increase in the p53-cAb-DNA concentration does not show uniformity in the increase in the fluorescence signal. It was observed that the 10 fmol/mL of p53-cAb-DNA along with any combination of p53-dAb-FB (0.015–0.35 fmol/mL) showed a linear increase in the fluorescence intensity. Therefore, the linearity coefficients for these measurements were determined. As depicted in Figure 2f (Figure S9), the linearity coefficient for the serial dilution of 5000 pg/mL of PIC using 0.07 fmol/mL anti-human-IgG-FB and 10 fmol/mL p53-cAb-DNA was found to be 0.992. Therefore, these concentrations of anti-human-IgG-FB and p53-cAb-DNA were considered optimum for PIC detection in plasma samples.

**Figure 3:**
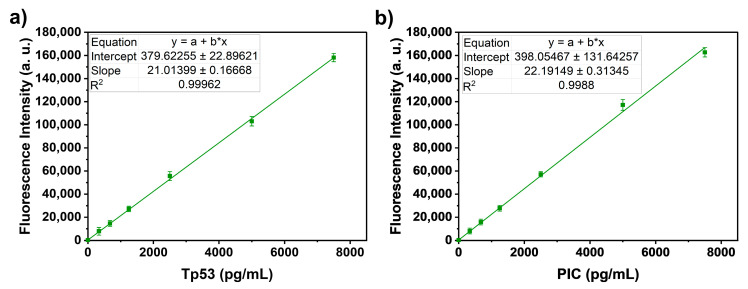
Standard curves for (**a**) Tp53 and (**b**) PIC detection.

The standard curves for the Tp53 and PIC detection were obtained using serial dilutions (0–7500 pg/mL) of the respective standard samples with the analyte-free human plasma. The mean of the fluorescence signals for eight calibration points were used to obtain the standard curves, as shown in Figure 3. The CLSI EP17-A2 guidelines were followed to determine the LOB and LOD for Tp53 and PIC detection [37]. The proposed LOB and LOD methods for Tp53 detection were 2.92 pg/mL and 7.41 pg/mL, respectively. The LOB and LOD methods for t PIC detection were 2.98 pg/mL and 5.74 pg/mL, respectively.

The linearity in the serial dilution test during biomarker detection using immunoassays confirms the accuracy of the measurements and the absence of the interference effect from the blood components [38]. Therefore, plasma samples from the healthy control group (n = 3) and lung cancer patients (n = 3) were used to evaluate the performance of the proposed method to detect Tp53 and PIC. As shown in Figures S10 and S11, the samples were serially diluted with the dilution factors ranging from 0.0625–1.0. The initial levels of Tp53 and PIC in plasma sample 1 (1132.2, pg/mL, 1480.9 pg/mL), sample 2 (4534.9 pg/mL, 5288.8 pg/mL), and sample 3 (626.1 pg/mL, 668.4 pg/mL) from the healthy control group and plasma sample 1 (1418.2 pg/mL, 3514.4 pg/mL), sample 2 (617.3 pg/mL, 1294.1 pg/mL), and sample 3 (670.0 pg/mL, 1741.4 pg/mL) from the lung cancer patients were measured using the respective standard curves. As shown in Figures S10 and S11, the linearity regression coefficients were 0.982–0.999 for all of the samples in the dilution range from 0.06 to 1.0 for healthy and lung cancer samples. These results indicate that the proposed method Tp53 and PIC detection demonstrates accuracy in the measurement and the absence of interference from the blood components.

The interference from blood components such as biotin, bilirubin, intra-lipid, and hemoglobin in immunoassays has been known for a long time [39]. Therefore, the plasma samples were spiked with the interfering materials to determine the interference of biotin, bilirubin, intra-lipid, and hemoglobin in Tp53 and PIC detection. The measurements were performed in the serially diluted samples. As shown in Figure S12, the plasma samples containing 1250 pg/mL Tp53 and 1250 pg/mL of PIC, respectively, were spiked individually with the biotin (3 μg/mL), bilirubin (0.2 mg/mL), intra-lipid (0.2%), and hemoglobin (1 mg/mL). Then, each sample was serially half diluted, and the fluorescence signal measurements for Tp53 and PIC detection were performed. The linearity regression coefficients were in the range of 0.990–0.995. These results indicate that the presence of biotin, bilirubin, lipid, and hemoglobin, even at levels that are several folds higher than their normal levels, do not interfere with Tp53 and PIC detection.

As shown in Table 1, we evaluated the clinical applicability of the presented method for Tp53 and PIC detection using 45 clinical samples. The samples from healthy individuals (n = 15) and lung cancer patients (n = 30) were used in this study. About 23 non-small cell lung cancer samples constituted stage I (n = 7), stage II (n = 6), stage III (n = 5), and stage IV (n = 5) lung cancer. The other seven samples were from patients with small-cell lung cancer comprising limited disease (n = 4) and extended disease (n = 3).

As shown in Table 1, the PIC levels were significantly higher in the lung cancer patients (310.3~7500.0 pg/mL) than they were in the healthy population (350.4~5676.3 pg/mL). In contrast, the Tp53 levels were substantially lower in the lung cancer patients (111.10~4530.6 pg/mL) than they were in the healthy population (271.0~4887.2 pg/mL). These results are in accordance with our initial hypothesis that the PIC levels and the Tp53 levels would be higher in lung cancer patients compared to healthy populations. Therefore, we also determined the PIC/Tp53 ratio and found that it was 2~3 times larger in lung cancer patients than in healthy people. Therefore, these values were used to determine the sensitivity and specificity of the proposed method for lung cancer diagnosis. The obtained results are presented in Table 2.

As presented in Table 2, the PIC or Tp53 alone is not efficient for the diagnosis of lung cancer, which si indicated by very low sensitivities of 16.7% and 23.3%, respectively. However, the use of the PIC/Tp53 ratio allows the detection of lung cancer with 78.7% sensitivity and a specificity of 93.3%. We recently reported a detection method for the CYFRA 21-1 antigen and CYFRA 21-1-Anti-CYFRA 21-1 autoantibody immune complex (CIC) [40]. The levels of these markers can be used to determine the CIC/CYFRA21-1 ratio, as shown in Table 2. Therefore, to increase the sensitivity of the presented method, we determined the cancer index using the following formula.

(1) $$Cancer\;Index=\frac{CS1}{CS2}\times \frac{LS1}{LS2}$$

where *CS*1, *CS*2, *LS*1, and *LS*2 are the PIC, Tp53, CIC, and CYFRA 21-1 levels in the plasma samples.

The determination of the cancer index in the plasma samples of healthy individuals and lung cancer patients allowed us to determine the sensitivity of 81.6% and the specificity of 93.3%. To the best of our knowledge, the sensitivity and specificity values presented here are much higher than the reported values.

Currently, LDCT is used as a standard method to screen lung cancer patients from the general population. However, LDCT has a very high false-positive rate. Thus additional biopsy-based tests are required to confirm lung cancer. Though biopsy is a well-known medical practice, it requires highly trained professionals, and patients suffer from various conditions, including discomfort and mental stress. Therefore, a biomarker-based lung cancer detection method can be a suitable companion to LDCT for lung cancer detection. In this regard, biomarkers such as the squamous cell carcinoma antigen (SCC Ag) and carcinoembryonic antigen (CEA) have also been scrutinized for their application in lung cancer diagnosis [41,42]. However, these biomarkers have very low sensitivity and specificity in lung cancer detection [43,44,45,46]. Thus, we believe that the initial findings presented in this manuscript for lung cancer detection with 81.6% specificity and 93.3% specificity using the Tp53 and PIC have high clinical significance. The number of clinical samples limits the results of this study. Thus, an additional clinical study with a more extensive sample set is required to assess the clinical applicability of the presented method.

## 4. Conclusions

Herein, we have developed a method for detecting and quantifying a p53-anti-p53–autoantibody complex and the total amount of p53 antigens (wild and mutant). The LOD for Tp53 and PIC detection were 7.41 pg/mL and 5.74 pg/mL, respectively. The detection ranges for both biomarkers were 0–7500 pg/mL. The known interfering agents in immunoassays such as biotin, bilirubin, intra-lipid, and hemoglobin did not show any effect on Tp53 and PIC detection, even at levels that were several folds higher than their normal levels. These results indicate that the presented method has high applicability in quantifying these biomarkers in the plasma samples of healthy and lung cancer patients. Furthermore, this study reports a preliminary investigation using the PIC/Tp53 ratio to detect stage I–IV lung cancers. The presented method detects lung cancers with 81.6% sensitivity and 93.3% specificity. These results indicate that the presented method has high applicability for the identification of lung cancer patients from the healthy population.

## Figures and Tables

**Table 1:** Characteristics of the study participants (n = 45).

Characteristic	Healthy Population (n = 15)	Lung Cancer Patients (n = 30)
Age, years (SD)	60 (58–68)	61.5 (47–81)
Male gender, n (%)	55 (64.5%)	17 (65.7%)
PIC, pg/mL (IQR)	2497.5 (350.4~5676.3)	2871.5 (310.3~7500.0)
Tp53, pg/mL (IQR)	2026.8 (271.0~4887.2)	1282.6 (111.10~4530.6)
PIC/Tp53 ratio (IQR)	1.23 (0.706~1.920)	2.22 (1.164~3.384)
CIC, pg/mL (IQR)	3.11 (0.411~8.146)	3.15 (0.499~8.038)
CYFRA 21-1, pg/mL (IQR)	2.13 (0.408~5.168)	1.35 (0.186~4.271)
CIC/CYFRA 21-1 ratio (IQR)	1.46 (0.411~8.146)	2.51 (1.632~4.244)
Cancer Index [(CS1/CS2) × (LS1/LS2)]	1.79 (0.759~2.766)	5.41 (3.615~9.401)

Tumor marker levels are presented as means and IQR. Counts are in numbers and percentages. CS1, PIC; CS2, Tp53; LS1, CIC; LS2, CYFRA 21-1.

**Table 2:** Results of the clinical study in terms of sensitivity and specificity for stage 0∼IV lung cancer detection (n = 45).

Biomarkers	Sensitivity (95% CI)	Specificity (95% CI)	PPV (95% CI)	NPV (95% CI)
PIC	16.7 (5.64~34.7)	93.3 (68.1~99.8)	83.3 (39.0~97.5)	35.9 (31.2~40.9)
Tp53	23.3 (9.93~42.3)	66.7 (38.4~88.2)	58.33 (34.8~78.6)	30.3 (22.42~39.6)
PIC/Tp53 ratio	78.7 (69.2~96.2)	93.3 (68.1~99.8)	96.3 (79.6~99.4)	77.8 (58.2~89.8)
CIC	13.3 (3.8~30.7)	86.7 (59.5~98.3)	66.6 (29.2~90.7)	33.3 (28.2~38.9)
CYFRA 21-1	10.0 (44.9~92.2)	73.3 (44.9~92.2)	42.9 (16.1~74.5)	28.9 (22.7~36.1)
CIC/CYFRA 21-1	73.3 (54.1~87.7)	93.3 (78.2~98.5)	76.7 (59.4–87.1)	65.2 (50.9~77.2)
Cancer Index [(CS1/CS2) × (LS1/LS2)]	81.6 (88.4~98.1)	93.3 (78.2~99.4)	86.7 (67.2–92.5)	71.2 (68.4–95.2)

## Data Availability

The data presented in this study are available upon request from the corresponding author.

## Supplementary Materials

The following are available online at https://www.mdpi.com/article/10.3390/bios12020127/s1, Figure S1: (a) p53 and mouse origin anti-53 detection antibody complex detection; (b) detection of PIC in lung cancer plasma sample; (c) standard curve using p53 and mouse origin anti-p53 detection antibody complex detection, Figure S2: Optimum incubation time for Tp53 detection. The bar graph for the fluorescence intensity and serial dilution (dilution factors: 1.0, 0.5. 0.25, 0.12, and 0.06) of a p53 (1250 pg/mL) from a standard sample at time intervals of 5, 0, 20, and 30 min is presented on the left side, and the corresponding linear curve-fitting results are presented on the right side. The linearity coefficient (R^2^ = 0.99701) for the 10 min incubation time was the highest, Figure S3: Optimum hybridization time for Tp53 detection. The bar graph for the fluorescence intensity and serial dilution (dilution factors: 1.0, 0.5. 0.25, 0.12, and 0.06) of a p53 (1250 pg/mL) from a standard sample at the time intervals of 5, 0, 20, and 30 min is presented on the left side, and the corresponding linear curve fitting results are presented on the right side. The linearity coefficient (R^2^ = 0.99057) for the 10 min incubation time was the highest, Figure S4: Optimum washing time for Tp53 detection. The bar graph for the fluorescence intensity and serial dilution (dilution factors: 1.0, 0.5. 0.25, 0.12, and 0.06) of a p53 (1250 pg/mL) from a standard sample at the time intervals of 5, 0, 20, and 30 min is presented on the left side, and the corresponding linear curve-fitting results are presented on the right side. The linearity coefficient (R^2^ = 0.99836) for the 10 min incubation time was the highest. Figure S5: Optimum incubation time for PIC detection. The bar graph for the fluorescence intensity and serial dilution (dilution factors: 1.0, 0.5. 0.25, 0.12, and 0.06) of a p53 (1250 pg/mL) of the standard sample at time intervals of 5, 0, 20, and 30 min is presented on the left side, and the corresponding linear curve-fitting results are presented on the right side. The linearity coefficient (R^2^ = 0.99449) for the 10 min incubation time was the highest, Figure S6: Optimum hybridization time for PIC detection. The bar graph for the fluorescence intensity and serial dilution (dilution factors: 1.0, 0.5. 0.25, 0.12, and 0.06) of a p53 (1250 pg/mL) from a standard sample at the time intervals of 5, 0, 20, and 30 min is presented on the left side, and the corresponding linear curve-fitting results are presented on the right side. The linearity coefficient (R^2^ = 0.99138) for the 10 min incubation time was the highest, Figure S7: Optimum washing time for PIC detection. The bar graph for the fluorescence intensity and serial dilution (dilution factors: 1.0, 0.5. 0.25, 0.12, and 0.06) of a p53 (1250 pg/mL) from a standard sample at the time intervals of 5, 0, 20, and 30 min is presented on the left side, and the corresponding linear curve-fitting results are presented on the right side. The linearity coefficient (R^2^ = 0.99643) for the 10 min incubation time was the highest, Figure S8: Linearity in the serial dilution test for Tp53 and PIC detection in three healthy control plasma samples (Sample 1, Tp53 = 1132.2 pg/mL, PIC = 1480.9 pg/mL; Sample 2, Tp53 = 4534.9 pg/mL, PIC = 5288.8 pg/mL; Sample 3, Tp53 = 626.1 pg/mL, PIC = 668.4 pg/mL), Figure S9: Linearity in the serial dilution test for Tp53 and PIC detection in three lung cancer plasma samples (Sample 1, Tp53 = 1418.2 pg/mL, PIC = 3514.4 pg/mL; Sample 2, Tp53 = 617.3 pg/mL, PIC = 1294.1 pg/mL; Sample 3, Tp53 = 670.0 pg/mL, PIC = 1741.4 pg/mL). Figure S10: Determination of biotin (3 μg/mL), bilirubin (0.2 mg/mL), intra-lipid (0.2%), and hemoglobin (1 mg/mL) interfance during the detection of Tp53 (1250 pg/mL) and PIC (1250 pg/mL) in spiked plasma samples.
