# Supplementary material for: Detection and Quantification of Tp53 and p53-Anti-p53 Autoantibody Immune Complex: Promising Biomarkers in Early Stage Lung Cancer Diagnosis
Source: Biosensors (Basel). 2022 Feb 16;12(2):127. doi: 10.3390/bios12020127 (PMC8870326; doi:10.3390/bios12020127)
Supplement: Supplementary file 1 [file biosensors-12-00127-s001.zip › biosensors-1585788-supplementary.pdf]

biosensors

MDPI

Supplementary

Detection and Quantification of Tp53 and p53-Anti-p53 Autoantibody Immune Complex: Promising Biomarkers in Early Stage Lung Cancer Diagnosis

Keum-Soo Song [1,†], Satish Balasaheb Nimse [2,†], Shrikant Dashrath Warkad [1], Jung-Hoon Kim [1], Hey-Jin Kim [3] and Taisun Kim [2,*]

[1] Biometrix Technology, Inc., 2-2 Bio Venture Plaza 56, Chuncheon 24232, Korea; hanlimsk@empas.com (K.-S.S.); shrikant.warkad@gmail.com (S.D.W.); jhkim@bmtchip.com (J.-H.K.)
[2] Institute of Applied Chemistry and Department of Chemistry, Hallym University, Chuncheon 200702, Korea; satish_nimse@hallym.ac.kr
[3] Department of Laboratory Medicine, Korea Cancer Center Hospital, Korea Institute of Radiological and Medical Sciences, Seoul 01812, Korea; heyjin@kirams.re.kr
* Correspondence: tskim@hallym.ac.kr
† Correspondence: These authors contributed equally to this work.

1. Quantification of PIC in the lung cancer sample using 9G DNAChip and DAGON method:

The quantification of PIC in a lung cancer sample using 9G DNAChip and DAGON method is explained here in brief as follows.

Citation: Song, K.-S.; Nimse, S.-B.; Warkad, S.-D.; Kim, J.-H; Kim, H-J; Kim, T-S. Detection and Quantification of Tp53 and p53-Anti-p53 Autoantibody Immune Complex: Promising Biomarkers in Early Stage Lung Cancer Diagnosis. *Biosensors* **2022**, *12*, 127. https://doi.org/10.3390/bios12020127

Received: 21 January 2022
Accepted: 14 February 2022
Published: 16 February 2022

Publisher's Note: MDPI stays neutral with regard to jurisdictional claims in published maps and institutional affiliations.

Copyright: © 2022 by the authors. Licensee MDPI, Basel, Switzerland. This article is an open access article distributed under the terms and conditions of the Creative Commons Attribution (CC BY) license (https://creativecommons.org/licenses/by/4.0/).

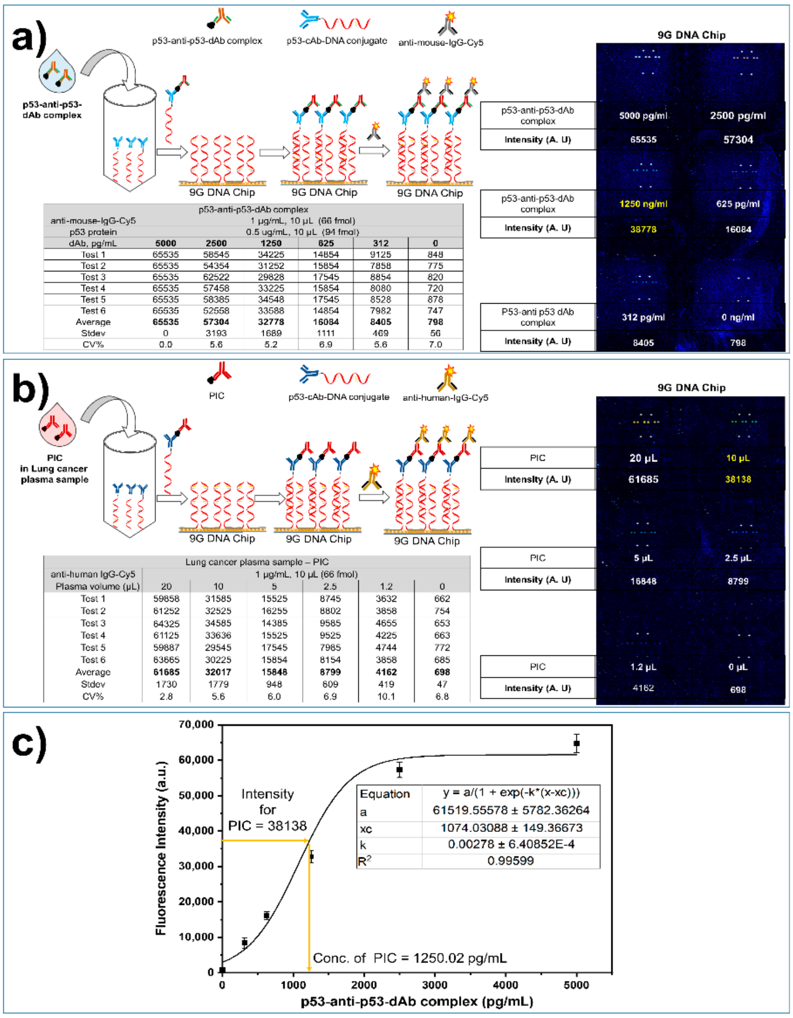

p53-anti-p53-dAb complex						
anti-mouse-IgG-Cy5	1 μg/mL, 10 μL (66 fmol)					
p53 protein	0.5 ug/mL, 10 μL (94 fmol)					
dAb, pg/mL	**5000**	**2500**	**1250**	**625**	**312**	**0**
Test 1	65535	58545	34225	14854	9125	848
Test 2	65535	54354	31252	15854	7858	775
Test 3	65535	62522	29828	17545	8854	820
Test 4	65535	57458	33225	15854	8080	720
Test 5	65535	58385	34548	17545	8528	878
Test 6	65535	52558	33588	14854	7982	747
Average	**65535**	**57304**	**32778**	**16084**	**8405**	**798**
Stdev	0	3193	1689	1111	469	56
CV%	0.0	5.6	5.2	6.9	5.6	7.0

Lung cancer plasma sample – PIC						
anti-human IgG-Cy5	1 μg/mL, 10 μL (66 fmol)					
Plasma volume (μL)	20	10	5	2.5	1.2	0
Test 1	59858	31585	15525	8745	3632	662
Test 2	61252	32525	16255	8802	3858	754
Test 3	64325	34585	14385	9585	4655	653
Test 4	61125	33636	15525	9525	4225	663
Test 5	59887	29545	17545	7985	4744	772
Test 6	63665	30225	15854	8154	3858	685
Average	**61685**	**32017**	**15848**	**8799**	**4162**	**698**
Stdev	1730	1779	948	609	419	47
CV%	2.8	5.6	6.0	6.9	10.1	6.8

Figure S1. a) p53 and mouse origin anti-53 detection antibody complex detection, b) Detection of PIC in lung cancer plasma sample, c) Standard curve using p53 and mouse origin anti-p53 detection antibody complex detection.

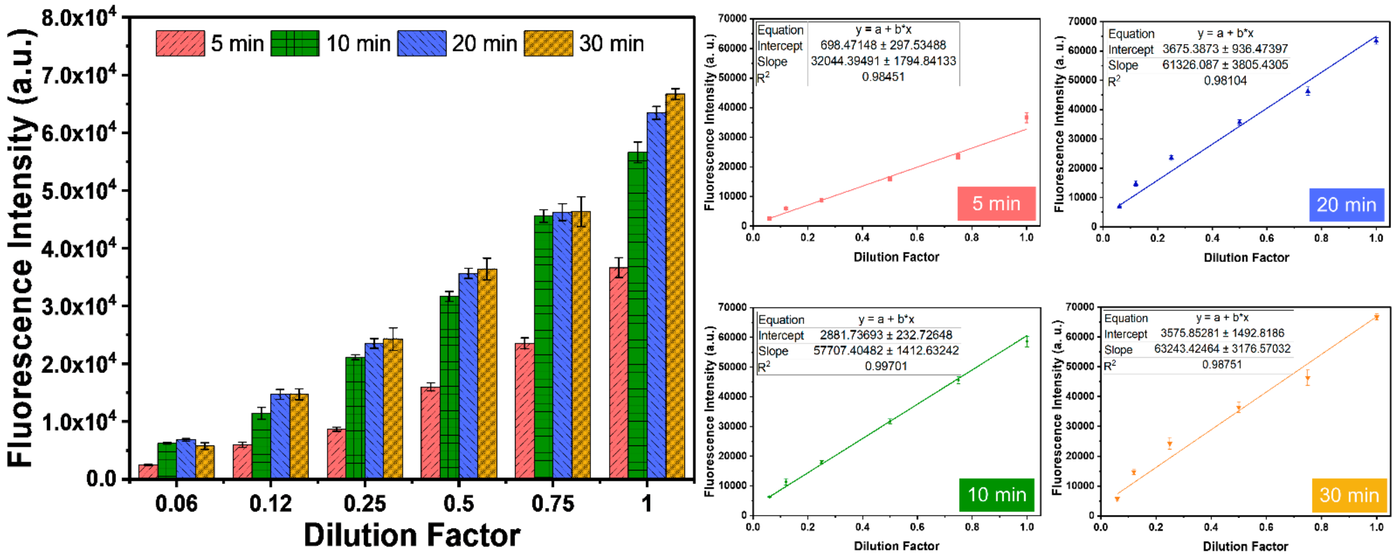

Figure S2. Optimum incubation time for Tp53 detection. The bar graph of fluorescence intensity and serial dilution (dilution factors: 1.0, 0.5. 0.25, 0.12, and 0.06) of a p53 (1250 pg/mL) of standard sample at time intervals of 5, 0, 20, and 30 min is presented on the left side and the corresponding

linear curve fitting results are presented on the right side. The linearity coefficient (R^2 = 0.99701) for the 10 min incubation time was highest.

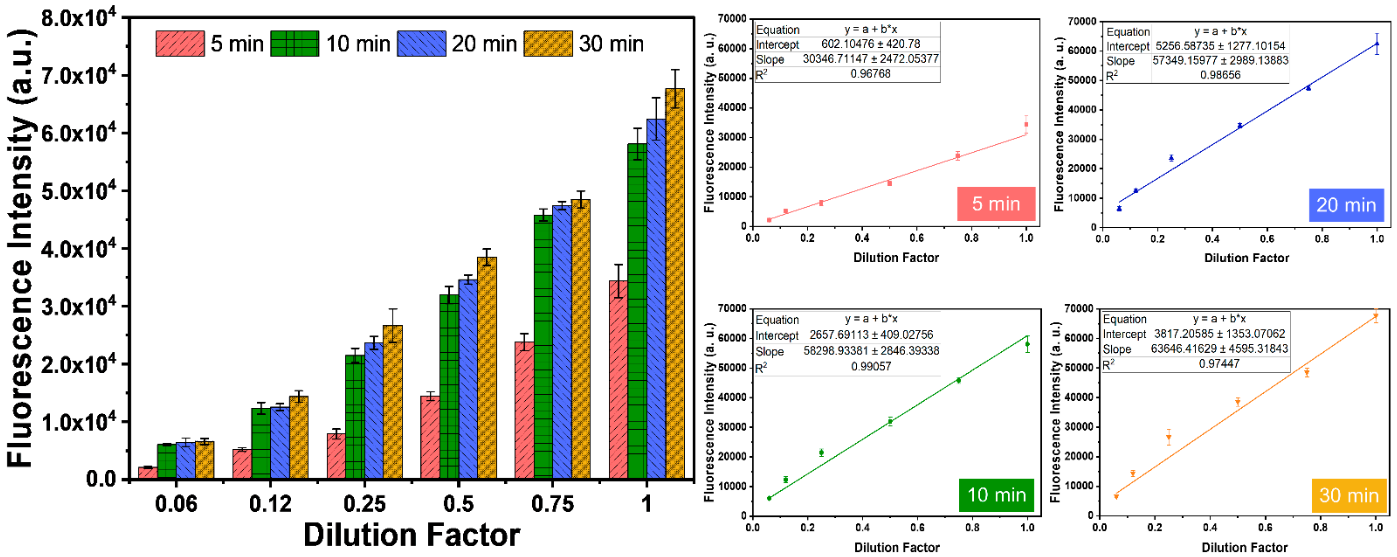

Figure S3. Optimum hybridization time for Tp53 detection. The bar graph of fluorescence intensity and serial dilution (dilution factors: 1.0, 0.5. 0.25, 0.12, and 0.06) of a p53 (1250 pg/mL) of standard sample at time intervals of 5, 0, 20, and 30 min is presented on the left side and the corresponding linear curve fitting results are presented on the right side. The linearity coefficient (R^2 = 0.99057) for the 10 min incubation time was highest.

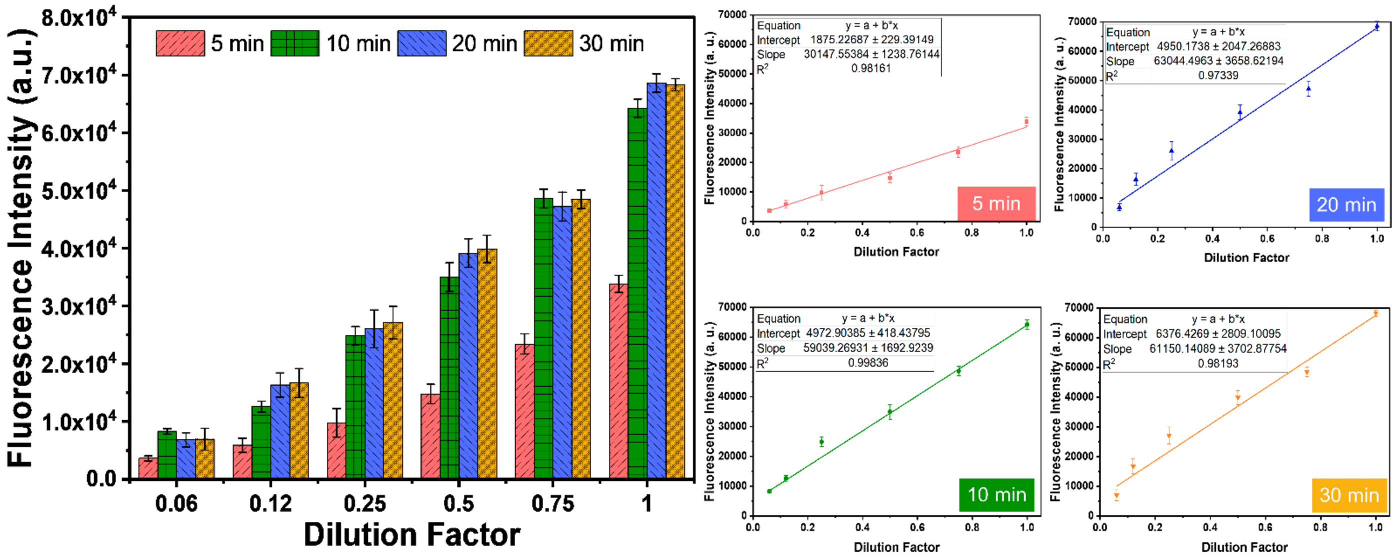

Figure S4. Optimum washing time for Tp53 detection. The bar graph of fluorescence intensity and serial dilution (dilution factors: 1.0, 0.5. 0.25, 0.12, and 0.06) of a p53 (1250 pg/mL) of standard sample at time intervals of 5, 0, 20, and 30 min is presented on the left side and the corresponding linear curve fitting results are presented on the right side. The linearity coefficient (R^2 = 0.99836) for the 10 min incubation time was highest.

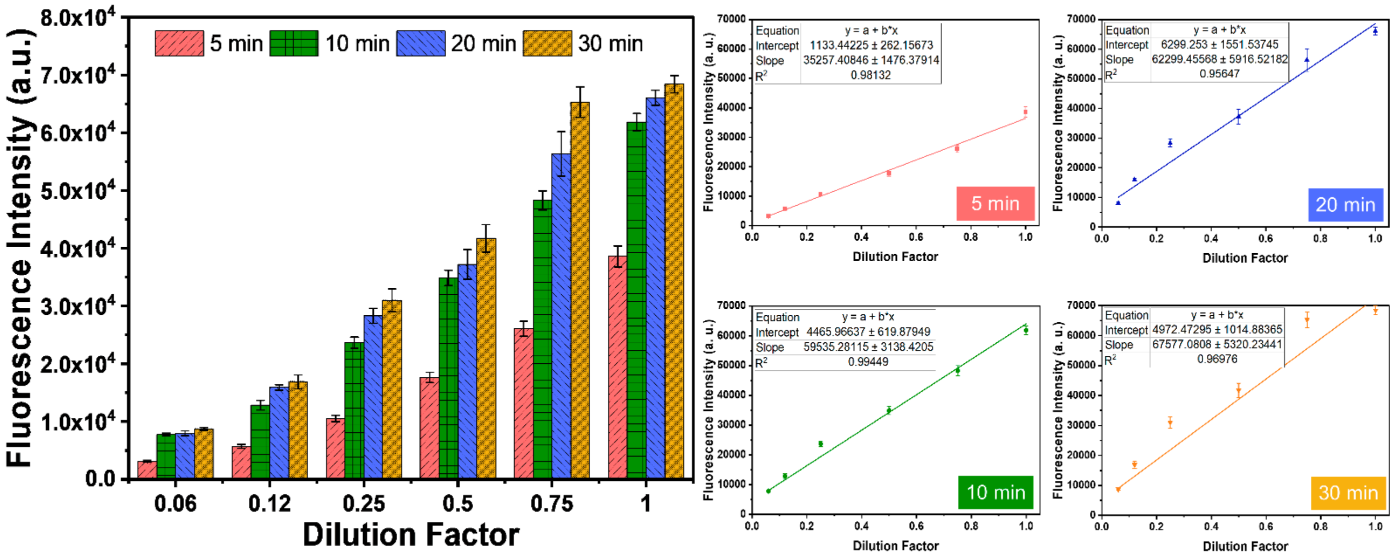

Figure S5. Optimum incubation time for PIC detection. The bar graph of fluorescence intensity and serial dilution (dilution factors: 1.0, 0.5. 0.25, 0.12, and 0.06) of a p53 (1250 pg/mL) of standard sample at time intervals of 5, 0, 20, and 30 min is presented on the left side and the corresponding linear curve fitting results are presented on the right side. The linearity coefficient (R^2 = 0.99449) for the 10 min incubation time was highest.

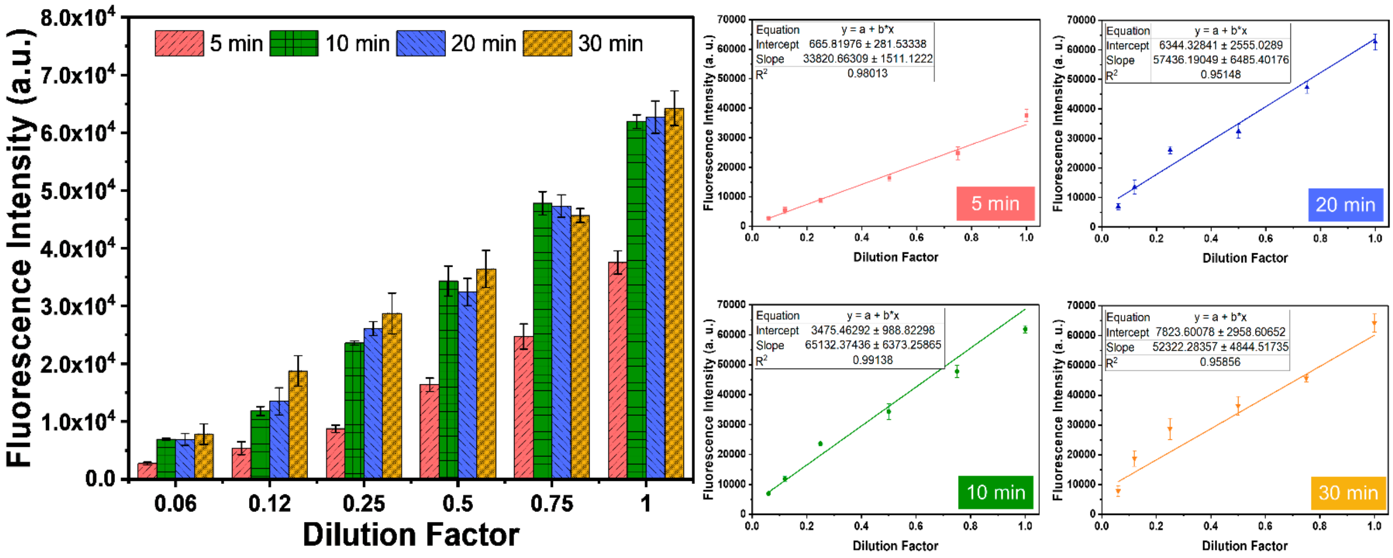

Figure S6. Optimum hybridization time for PIC detection. The bar graph of fluorescence intensity and serial dilution (dilution factors: 1.0, 0.5. 0.25, 0.12, and 0.06) of a p53 (1250 pg/mL) of standard sample at time intervals of 5, 0, 20, and 30 min is presented on the left side and the corresponding linear curve fitting results are presented on the right side. The linearity coefficient (R^2 = 0.99138) for the 10 min incubation time was highest.

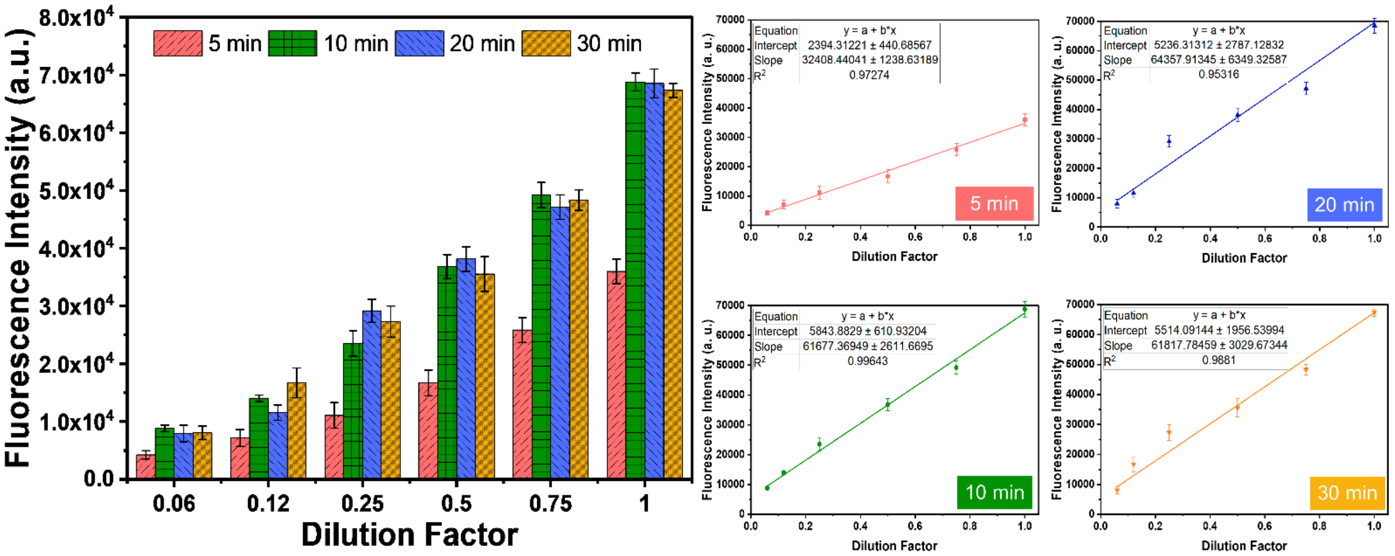

Figure S7. Optimum washing time for PIC detection. The bar graph of fluorescence intensity and serial dilution (dilution factors: 1.0, 0.5. 0.25, 0.12, and 0.06) of a p53 (1250 pg/mL) of standard sample at time intervals of 5, 0, 20, and 30 min is presented on the left side and the corresponding linear curve fitting results are presented on the right side. The linearity coefficient (R^2 = 0.99643) for the 10 min incubation time was highest.

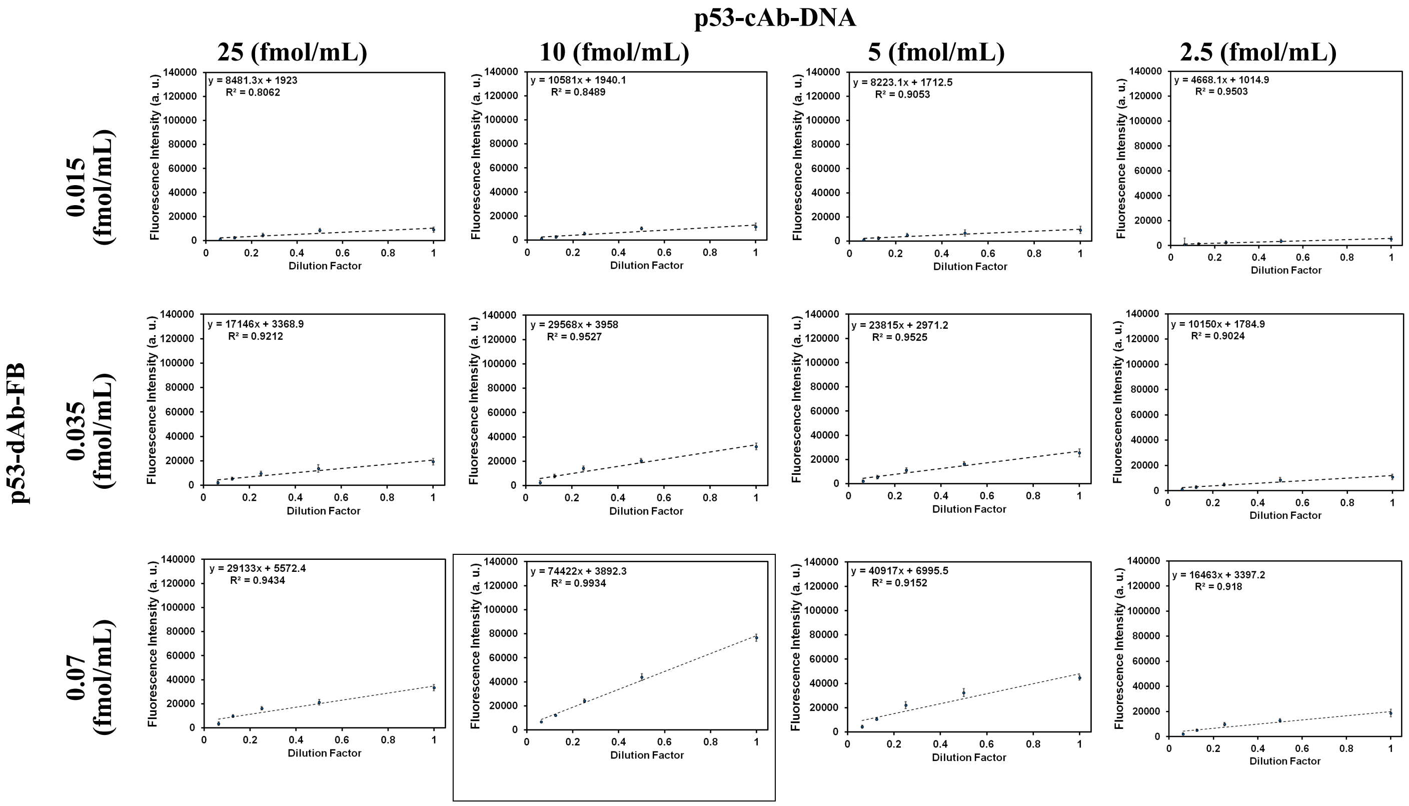

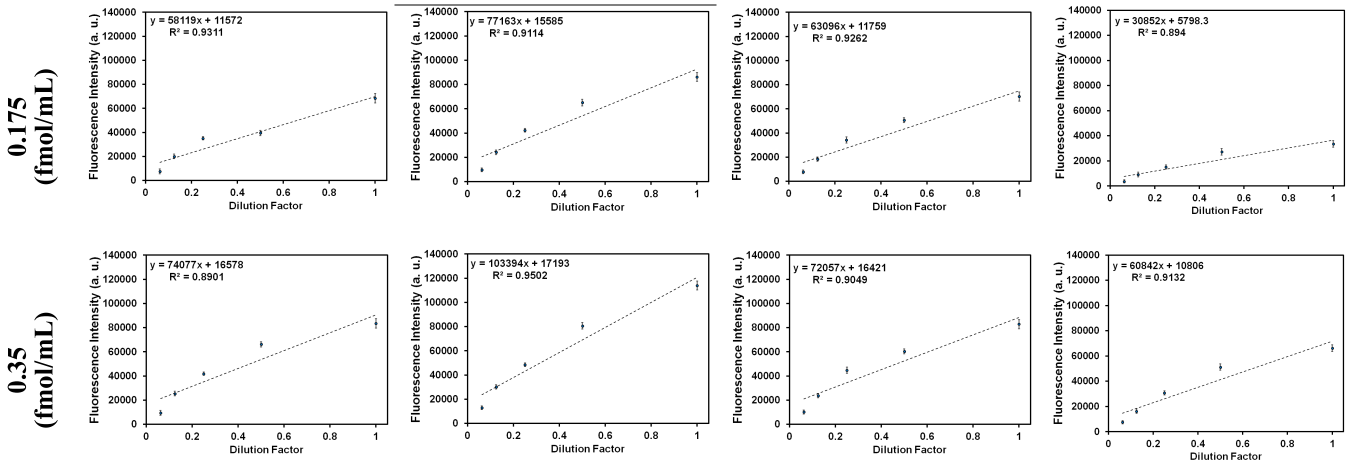

Figure S8. Linearity in the serial dilution test for 5000 pg/mL of Tp53 using 0.35, 0.175, 0.07, 0.035, 0.015 fmol/mL of p53-dAb-FB and 25, 10, 5, 2.5 fmol/mL of p53-cAb-DNA.

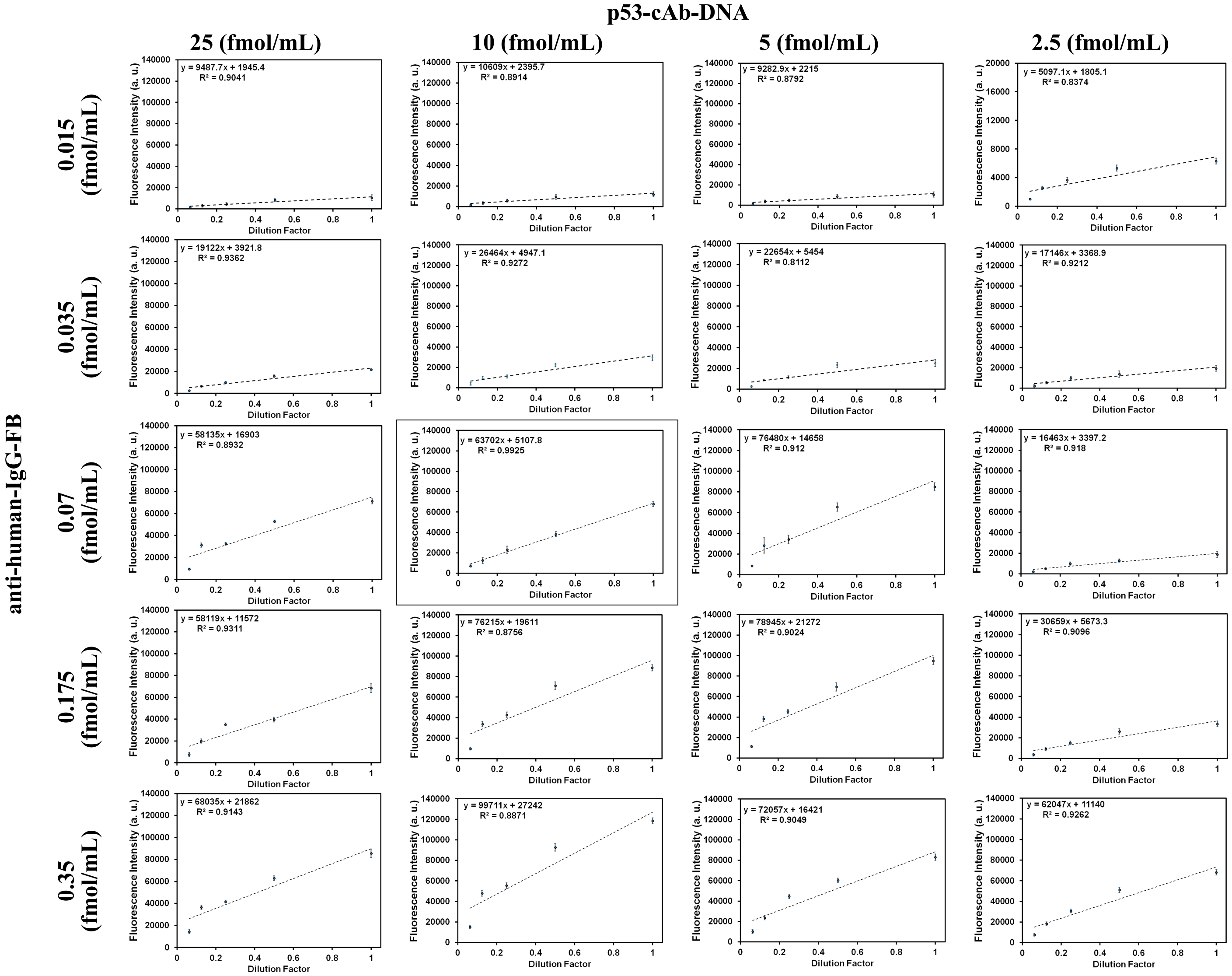

Figure S9. Linearity in the serial dilution test for 5000 pg/mL of PIC using 0.35, 0.175, 0.07, 0.035, 0.015 fmol/mL of anti-human-IgG-FB and 25, 10, 5, 2.5 fmol/mL of p53-cAb-DNA.

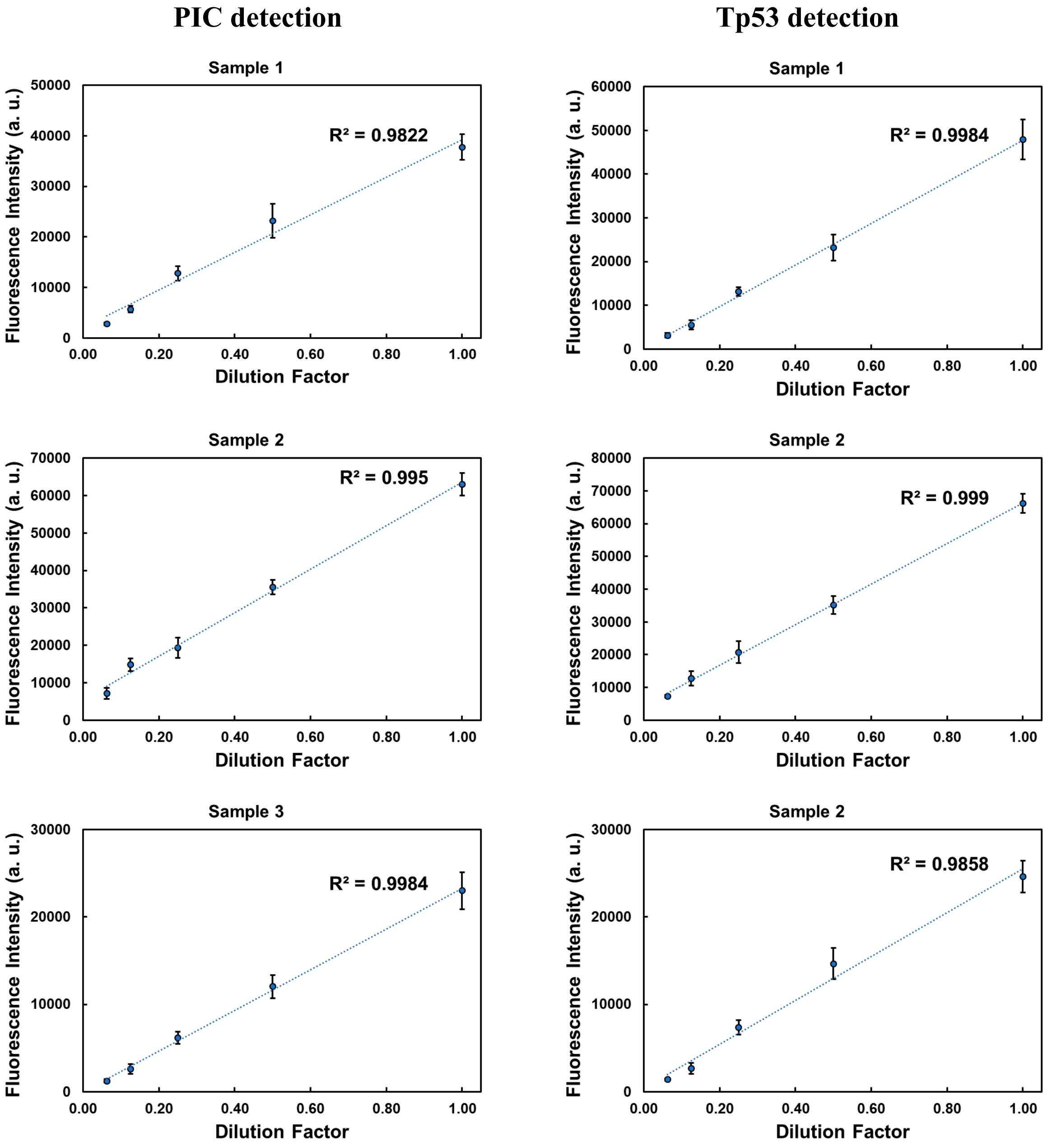

Figure S10. Linearity in the serial dilution test for Tp53 and PIC detection in three healthy control plasma samples (Sample 1, Tp53 = 1132.2 pg/mL, PIC = 1480.9 pg/mL; Sample 2, Tp53 = 4534.9 pg/mL, PIC = 5288.8pg/mL pg/mL; Sample 3, Tp53 = 626.1 pg/mL, PIC = 668.4 pg/mL).

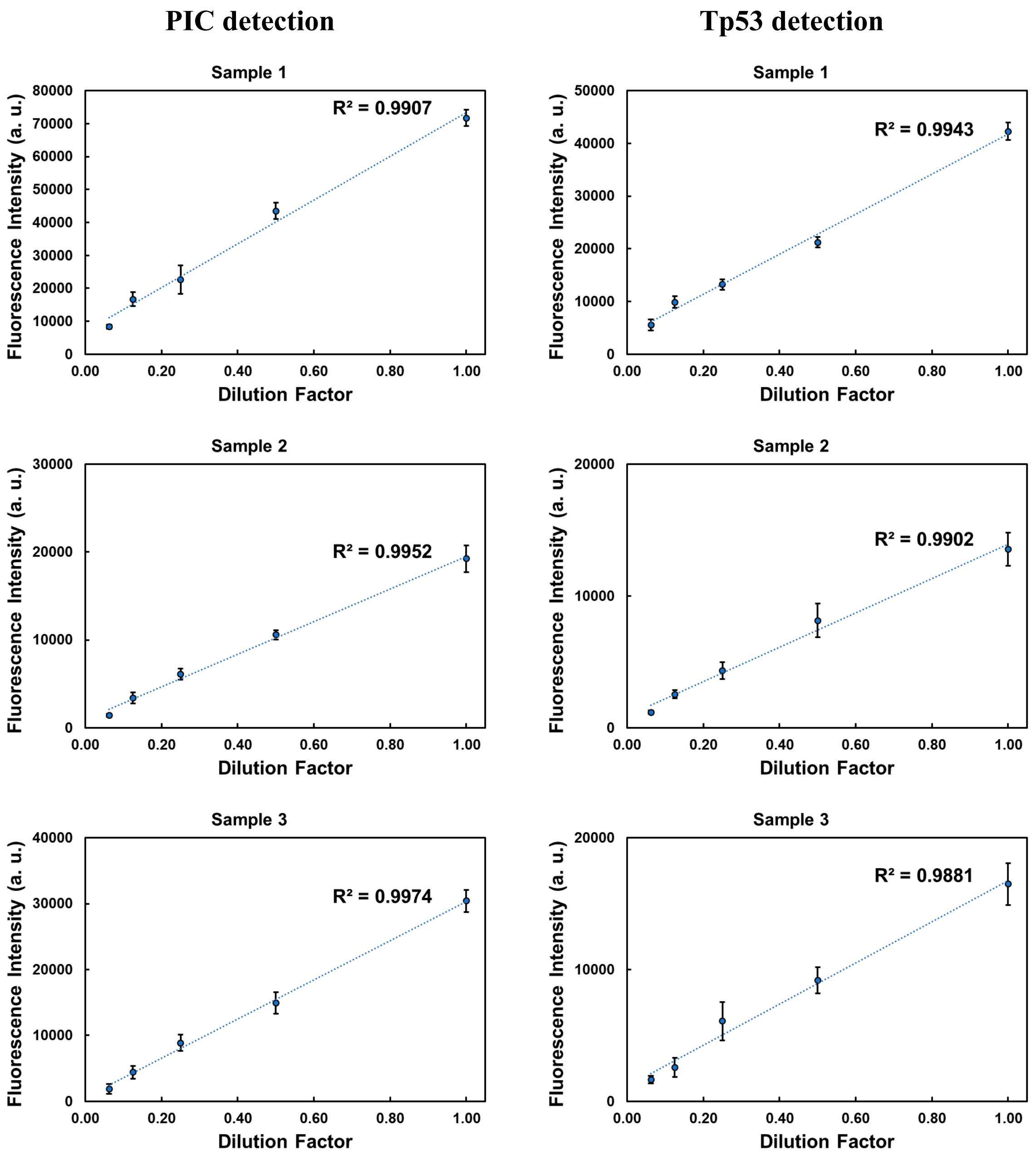

Figure S11. Linearity in the serial dilution test for Tp53 and PIC detection in three lung cancer plasma samples (Sample 1, Tp53 = 1418.2 pg/mL, PIC = 3514.4 pg/mL; Sample 2, Tp53 = 617.3 pg/mL, PIC = 1294.1 pg/mL; Sample 3, Tp53 = 670.0 pg/mL, PIC = 1741.4pg/mL).

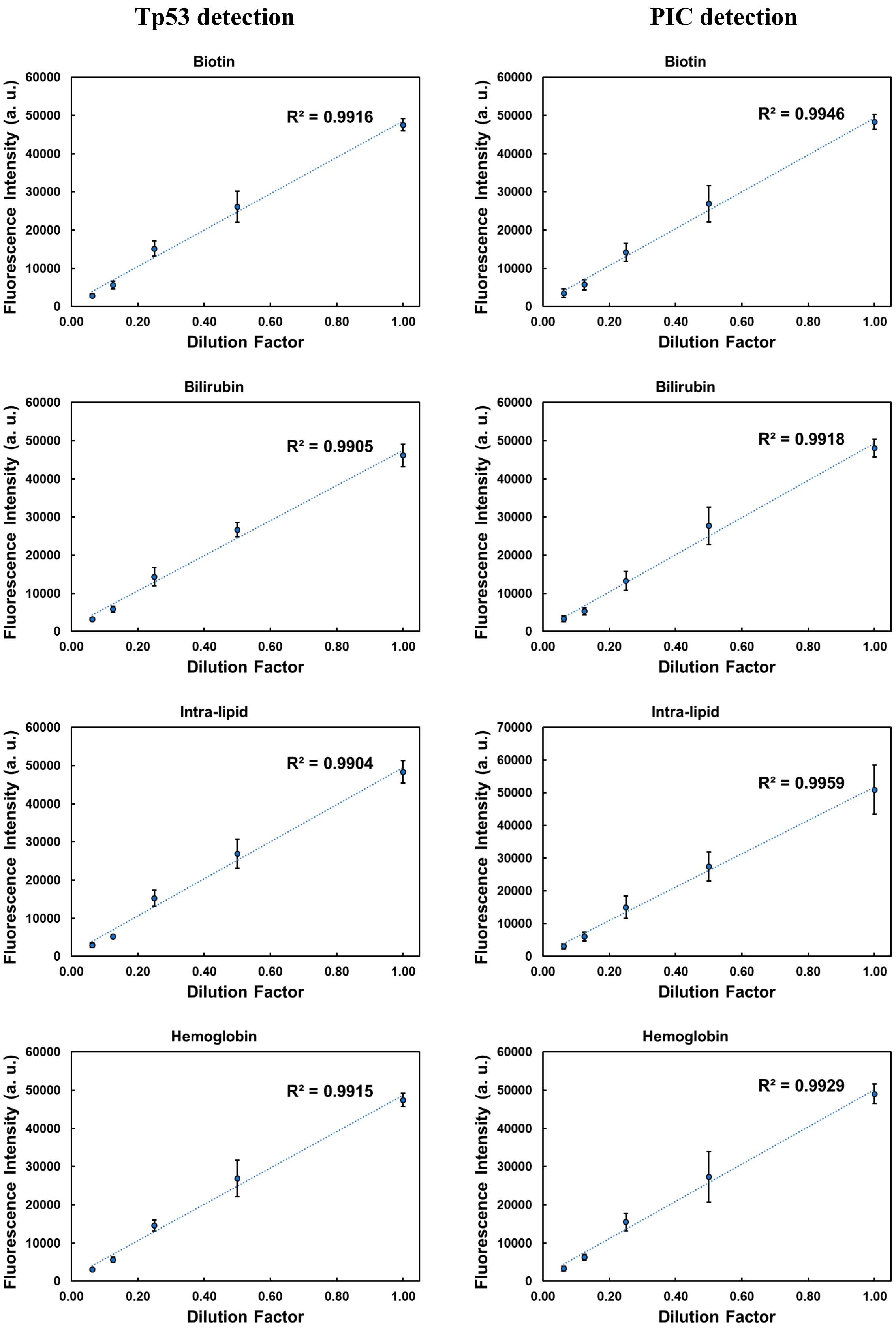
Tp53 detection
PIC detection
Biotin
R² = 0.9916
Biotin
R² = 0.9946
Bilirubin
R² = 0.9905
Bilirubin
R² = 0.9918
Intra-lipid
R² = 0.9904
Intra-lipid
R² = 0.9959
Hemoglobin
R² = 0.9915
Hemoglobin
R² = 0.9929
Fluorescence Intensity (a. u.)
Dilution Factor
0.00
0.20
0.40
0.60
0.80
1.00
0
10000
20000
30000
40000
50000
60000
70000

Figure S12. Determination of interference of biotin (3 μg/mL), bilirubin (0.2 mg/mL), intra-lipid (0.2%), and hemoglobin (1 mg/mL) on the detection of Tp53 (1250 pg/mL) and PIC (1250 pg/mL) in spiked plasma samples.
